# Improved mitochondrial function corrects immunodeficiency and impaired respiration in neonicotinoid exposed bumblebees

**DOI:** 10.1371/journal.pone.0256581

**Published:** 2021-08-26

**Authors:** Michael Barry Powner, Graham Priestley, Chris Hogg, Glen Jeffery

**Affiliations:** 1 Centre for Applied Vision Research, City University of London, London, United Kingdom; 2 Clinical Forums Ltd, Chichester, United Kingdom; 3 Institute of Ophthalmology, University College London, London, United Kingdom; University of Alberta, CANADA

## Abstract

Neonicotinoid pesticides undermine pollinating insects including bumblebees. However, we have previously shown that mitochondrial damage induced by neonicotinoids can be corrected by 670nm light exposure. But we do not know if this protection extends to immunity or what the minimum effective level of 670nm light exposure is necessary for protection. We use whole body bee respiration *in vivo* as a metric of neonicotinoid damage and assess the amount of light exposure needed to correct it. We reveal that only 1 min of 670nm exposure is sufficient to correct respiratory deficits induced by pesticide and that this also completely repairs damaged immunocompetence measured by haemocyte counts and the antibacterial action of hemolymph. Further, this single 1 min exposure remains effective for 3–6 days. Longer exposures were not more effective. Such data are key for development of protective light strategies that can be delivered by relatively small economic devices placed in hives.

## Background

Bee populations are declining worldwide [1–5] impacting ecosystem stability and pollination [6–11]. Factors suspected to contribute to this decline include parasites and pathogens [12], pesticide exposure [13, 14], habitat loss, fragmentation [15], and climate change [16]. However, neonicotinoid insecticides are key players [17–21]. They undermine mitochondrial function by overstimulating acetylcholine receptors [22, 23]. Neonicotinoids produce a sustained influx of Ca^2+^ ions into neurons that is associated with increased reactive oxygen species production by mitochondria [24]. Mitochondrial changes also occur because neonicotinoids inhibit complex III and IV respiration [25]. They also alter gene expression [26] and affect thermoregulation [21, 27]. The consequence is bumblebees have reduced ATP production, impaired vision, memory, and restricted mobility resulting in reduced feeding leading to death [28–35]. However, additionally neonicotinoids also suppress bee immunity rendering them vulnerable to pathogens [34, 36–38] and this may be a significant independent factor.

Reduced mitochondrial function can be increased by long wavelength light. In diverse species this improves membrane potential and compromised ATP production [39–41]. Mitochondria do not appear to absorb these wavelengths directly [42, 43]. Rather, the mechanism of action is currently thought to be related to light absorption by the nanoscopic interfacial water layer around mitochondrial ATP rotor pumps that reduces its viscosity and allows these to achieve greater momentum. In support of this there is marked overlap between patterns of water absorption of longer wavelengths and improved mitochondrial function [44]. In bumblebees and *Drosophila*, it also improves retinal function, mobility, memory and extends average lifespan [33, 45, 46]. 670nm light protects bumblebees from Imidacloprid, a major neonicotinoid pesticide, when given for 15 mins twice daily over 10 days. [33]. But to be used effectively against pesticide a greater understanding of its overall impact is needed. Particularly how much light is required and if it has influence over immunocompetence.

Hence, we determine the influence of 670 nm exposure (photobiomodulation) on pesticide treated bees by measuring whole-body respiration and ascertaining minimum light exposure times to correct deficits. Further, we asked how long minimal exposures remained protective. We then used these data to correct induced deficits in immunocompetence. We reveal that only single brief exposures are needed. This provides a realistic opportunity to translate 670nm exposure into the field to protect bee populations.

## Materials and methods

### Animals

Bumblebees (*Bombus terrestr*is) colonies were obtained from Koppert UK. Three colonies were studied, each experimental group containing replicates from each colony, kept at the same ratios. Experiments were undertaken in summer months. Bumblebees were maintained *ad libitum* on 50% sucrose solution in water and pollen. Where bees were exposed to Imidacloprid it was commonly for 5 day prior to an experiment, however full details are given under each heading below and in the Figure legends.

### Exposure to Imidacloprid and/or 670nm light

Bumblebees were transferred from colonies and placed in 3L (190 x 143 x 120 mm) transparent plastic containers under standard 12/12 light dark cycles, approximately eight bees per box, separate colonies were not mixed. Room illumination was indirect, and the spectrum shows minimal 670nm content, further details of the room lighting, including the full spectrum can be found in Begum et al., see their Fig 2 [39]. Bumblebees were exposed to a field representative concentration of Imidacloprid, 10nM, (2.56 ppb, estimation based on concentration) [47, 48]. Imidacloprid was given in 50% sucrose solution in water, *ad libitum* feeding throughout each experiment. 670nm was delivered by specific 670nm light emitting LEDs. The spectral composition and energy output of these were checked before and after use. The energy levels in light exposures were a total of 40mw/cm^2^ from two light sources at either end of the container.

Intervention comparisons experiments had 4 groups; control, Imidacloprid, Imidacloprid + 670nm light and 670nm light on its own, identical to Powner et al. [33]. Here, 670nm light exposure was given twice daily by illumination from above with each exposure being 15 mins, and 12 h between exposures. First 670nm exposure: 1h after transfer into 3L containers and potential exposure to Imidacloprid. Those treated with Imidacloprid were exposed continuously throughout via the sugar water, for 5 days.

To determine how long 670nm light exposure was needed to induce a positive biological response, and once withdrawn how long its positive impact lasted, a time series of experiments were undertaken. Here bumblebees were again transferred from colonies and placed in 3L transparent plastic containers under standard 12/12 light dark cycles. Those exposed to Imidacloprid were given 10nM Imidacloprid in 50% sucrose solution. Individual bumblebees were subsequently exposed to specific durations of red light after Imidacloprid exposure (or control). Five days after experimental diet onset, single bumblebees were isolated into clear 12 ml plastic test tubes, sealed in the tube using a gas permeable plastic bung. 670nm LED devices were placed either side of the test tube to deliver the required 670nm duration, at 40 mW/cm^2^, which did not increase local temperature. Bumblebees had space to move up/down within the field of light. Bumblebees were returned to their 3L containers and respiration or immunocompetency experiment protocols followed.

### Measuring individual bumblebee respiration

This was undertaken to determine the impact of Imidacloprid on whole body respiration and how this was altered by 670nm light or immune challenge.

#### Respiration rate measurements

**Fig 1:**
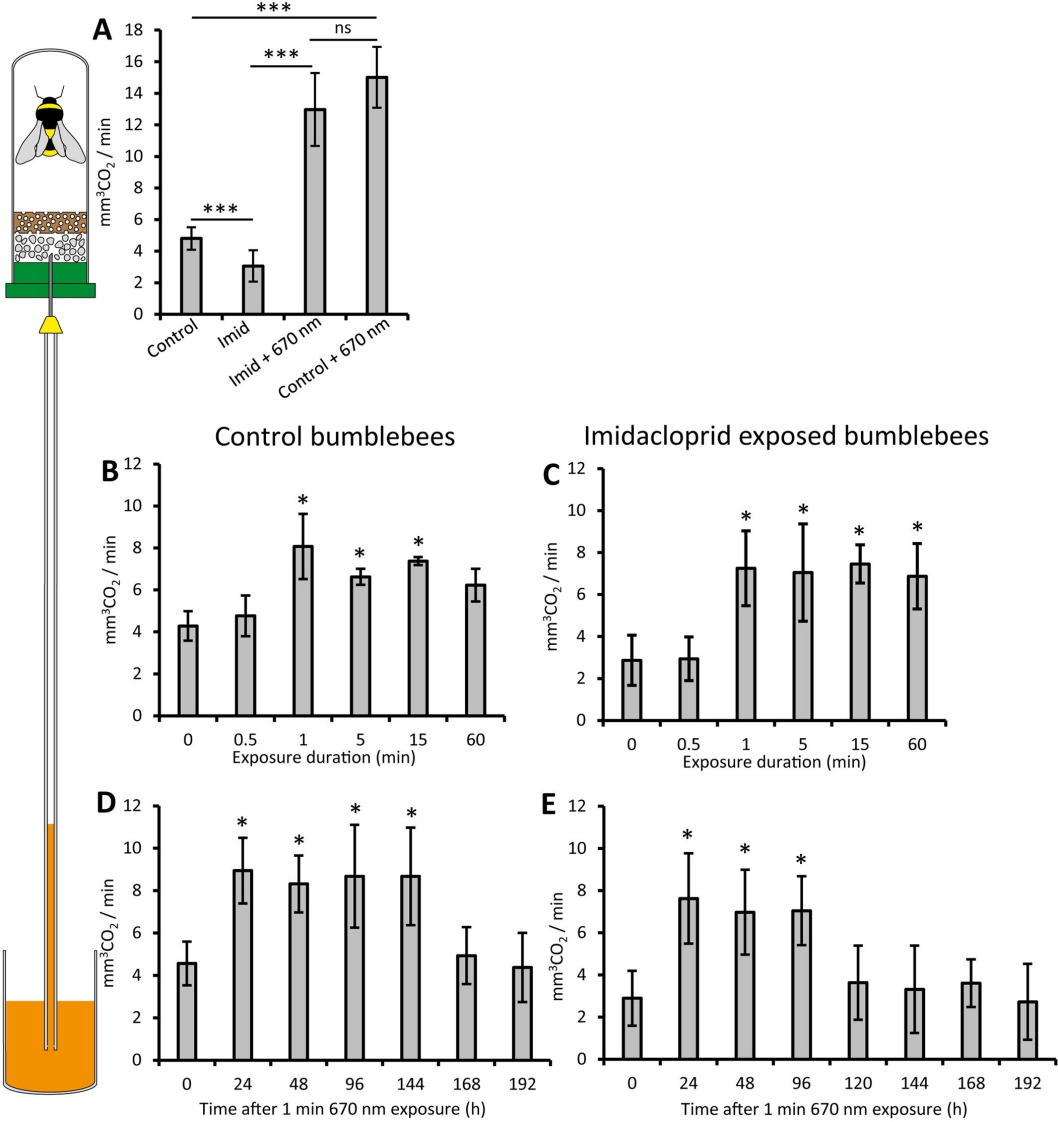
Effects on respiration. Individual bumblebee respiration was measured as shown in the schematic to the left. Bumblebees were subjected to 5 day exposure to Imidacloprid and/or 670nm (≥ 18 bees per group). A. Imidacloprid had a significant impact reducing respiration, but this was corrected by exposure to 670nm light. The amount of light required, and the duration of this effect were then determined. B. Bumblebees (≥ 20 bees per group) were exposed to 670nm light for 0.5, 1, 5, 15 or 60mins and respiration measured. Controls (0) were not exposed. Exposure for 0.5 min had no impact. However, 1 min light exposure significantly increased respiration. Longer exposures were similar. The same metric was determined in Imidacloprid exposed bumblebees, again 0.5min exposure had no impact, however durations between 1 and 60mins induced significant increase in respiration (C). Bumblebees (≥ 18 bees per group) were exposed to a single 670nm for 1 min (D) and respiration measured at 24h intervals. This elevated respiration for 144h but then declined rapidly. A single 1min exposure of 670nm in Imidacloprid treated bees also elevated respiration, for 96h (E), a slight reduction in duration compared to the effect of 670nm on non-pesticide exposed bees (D). Abbreviations: *; p < 0.05, ***; p < 0.005, ns; no significance. Imid; Imidacloprid.

Whole body respiration was measured using a protocol adapted from Yatsenko et al. [49]. In brief, bumblebees were individually transferred into 12 ml plastic test tubes that were transparent to 670nm light. They were sealed in the tube using a gas permeable plastic bung. Soda lime was placed within the tube and the tube sealed with an airtight rubber bung. Sealed tubes had a syringe needle (19 gauge) forced through the base connected to a length of 1mm internal diameter clear tubing, the other end of which was placed in an ink bottle (See schematic illustration in Fig 1). This was identical to the procedure used by Weinrich et al. (See their Fig 2) [50]. In this sealed environment, when bumblebees expired CO_2_ it was absorbed by the soda lime generating a drop in internal pressure that could be measured by the volume of ink passing up the tube. Respiration was monitored this way over 1 h, and the average respiration rate per min calculated.

**Fig 2:**
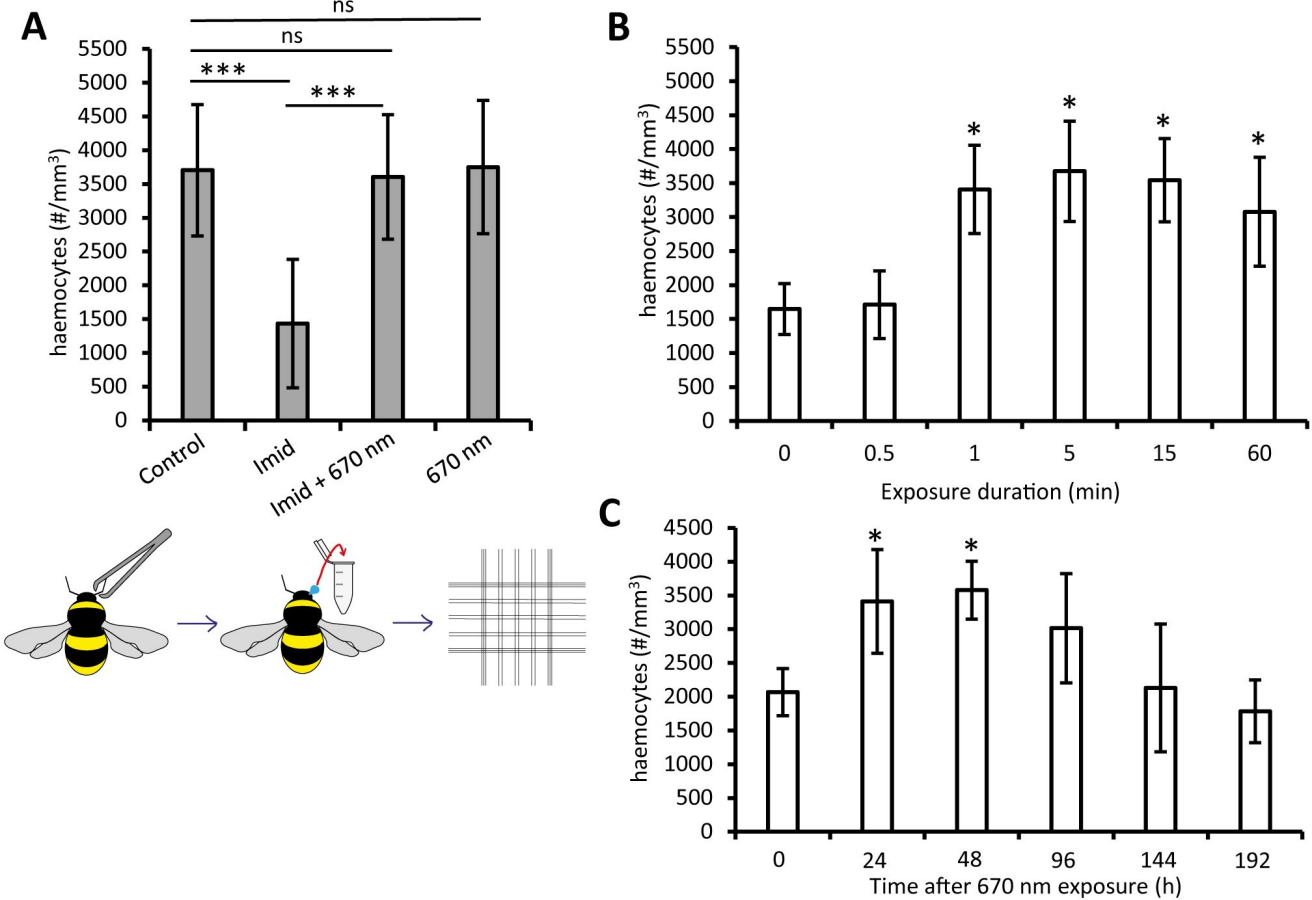
Bumblebees were subjected to 5 day exposure to Imidacloprid and/or/none twice daily 670 nm. Haemolymph was collected from antenna base and transferred to a haemocytometer as shown in the schematic (lower left). Haemocyte counts were used as a metric of immune health. Bumblebees were either exposed to Imidacloprid and or 670nm light. Exposures were for 5 days. Imidacloprid reduced haemocyte numbers (A), which was corrected with twice daily exposure to 670nm light (A). As with respiration we then asked what 670nm duration was needed for correction B. Bumblebees (≥ 20 bees per group) maintained on Imidacloprid were exposed to 670nm light for 0.5, 1, 5, 15 or 60mins. Controls (0) were unexposed. Exposure for 0.5 min had no impact, but 1 min exposure significantly increased haemocyte numbers. Longer exposures were similar. Bumblebees (≥ 22 bees per group) were exposed to a single 670nm for 1 min (C) and haemocytes counted at 24h intervals to determine duration of effect (C). (≥ 20 bees per group); 1 min exposures again restored haemocyte counts (D) over 48h (E), however, a decrease was observed from 96h post exposure (E), with no significance difference from the control observed afterwards. Abbreviations: *; p < 0.05. **; p < 0.01, ***; p < 0.005, ns; no significance, Imid; Imidacloprid.

To compare the effect that 670nm light has upon control and Imidacloprid exposed bees, the intervention comparison setup from above was used. Bumblebees were subjected to 5 day exposure to Imidacloprid and/or 670nm light. Respiration rates were monitored 12 h after the last 670nm light exposure.

To determine how long a period of 670nm light exposure was needed to improve respiration, and once withdrawn, how long its positive impact lasted, a time series of experiments were undertaken. Progressive exposures of increasing duration of 670nm were given at 0, 0.5, 1, 5, 15 and 60mins and respiration monitored in real time post 670nm exposure. To determine how long the positive impact of the 670nm light exposure remained effective, bumblebees were exposed to the minimal exposure period that was significantly improved respiration and then returned to the 3L containers and removed for respiration experiments at time periods of 0, 24, 48, 96, 144, 168 or 192 h. Each group of bumblebees measured at each time interval were independent from each other, bees were culled after the respiration rate was measured.

### Determining bumblebee immune system health

The following were undertaken to determine the impact of Imidacloprid on immunocompetency and how this was altered by 670nm light.

#### Haemolymph collection

Haemolymph was collected using the protocol of Borsuk et al. [51]. Briefly, heads of bumblebees were swabbed with 70% ethanol and left to evaporate. An antenna of the bumblebee was detached, and haemolymph outflow induced by pressing the abdomen (See schematic illustration in Fig 2A). A bead (~5 μl) of haemolymph formed at the antenna base and collected with a pipette. This was immediately transferred to an Eppendorf and kept in ice to prevent melanisation. For haemocyte counts, the haemolymph was used immediately. Haemolymph destined for inhibition zone assays was stored at -80°C for 1 week until the assay was performed.

#### Total haemocyte count

1 μl of haemolymph was transferred to an Eppendorf tube containing 4 μl phosphate buffered saline and stored on ice. Diluted haemolymph was transferred to a haemocytometer (Bürker chamber); haemocytes were counted (an average of three chambers per bumblebee were recorded) under an inverted phase contrast/fluorescent microscope. Hoechst staining was used to confirm nucleus presence and cellular identity.

670nm light effect upon control and Imidacloprid exposed bumblebees was determined using the intervention comparison setup followed by total haemocytes counted. Mirroring the timeline for respiration.

To determine how long a period of 670nm light exposure was needed to increase total haemocyte counts, and once withdrawn, how long its positive impact lasted, the same time series of experiments were undertaken as for respiration, using progressive exposures of increasing duration of 670nm, and to determine how long the positive impact of the 670nm light exposure remained effective, bumblebees were exposed to the minimal exposure period that was significantly improved haemocyte numbers (1 min), returned to the 3L containers and haemocyte counts determined at time periods of 0, 24, 48, 96, 144 or 192 h.

#### Inhibition-zone assay

Following 4 day exposure to Imidacloprid and/or/neither 670nm, the immune system of the bumblebee was challenged by the injection (Hamilton syringe) into the abdomen of 1 μl of heat-inactivated (90°C, 5 min) *Escherichia coli* (OD 0.5), suspended in sterile bee-Ringer solution [52]. Heat-inactivation of bacteria was confirmed by plating an aliquot of suspension onto agar medium and observation of no colony growth. Bumblebees were immobilised on ice for 5 minutes prior to injection. After 24 h haemolymph was collected as previously described (now on day 5 total exposure to varied conditions, respectively). Agar plates (9 cm diameter) containing LB medium were spread with 0.2 ml of fresh overnight cultures of *Micrococcus flavus* bacteria (OD 0.5). 1.5 μl aliquots of haemolymph were pipetted onto sterile 3 mm discs of blotting paper on the surface of the agar plate. The plates were inverted and incubated at 37°C for 24 h and the diameter of each inhibition zone was measured using digital Vernier calipers. Two diameter measurements, perpendicular to each other were taken and averaged. Each bee sample was tested in duplicate, and the mean used for analysis (See schematic illustration in Fig 2).

### Statistical analysis

Kruskal-Wallis H test and Mann Whitney U test were used to assess significance between groups. The Bonferroni correction for multiple comparisons was applied. Error bars are Standard Error of the Mean (SEM).

## Results

### Respiration

Imidacloprid undermines mitochondrial function and likely impacts on respiration. We quantify whole body respiration in individual bumblebees by measuring CO_2_ production in normal bees and those exposed to Imidacloprid over 5 days. The rationale for 5 days was for consistency with our previous study [33]. First, we confirmed that Imidacloprid significantly reduced respiration by 36%, which was corrected with 670nm light given for 15 mins twice daily (Fig 1A).

The light produced an enhanced respiratory response that from Powner *et al*. is associated with improved metrics including mean lifespan (Fig 1A) [33]. Light exposure length was then reduced to determine the minimal amount needed for protection. Single 670nm exposures from 0.5 to 1, 5, 15 and 60mins were given. In both normal and pesticide treated bumblebees 0.5 mins exposure had no impact. In healthy bumblebees, 1 min exposure increased respiration significantly, but longer times did not result in a further increase (Fig 1B and 1C). Elevated respiration from single exposures in healthy bees lasted 144h (Fig 1D). Similar patterns were found in Imidacloprid treated bees; however, the effective period of protection was reduced to 96h (Fig 1E). Hence, there is no evidence for exposures longer than 1min being more effective.

### Immunocompetence

Cellular energy demand increases in immune responses. Haemocytes play a key role in the invertebrate immune system. Total haemocyte counts [53, 54], and the haemolymphs ability to inhibit bacterial activity [36] are metrics of immunocompetence, both of which are compromised in Imidacloprid exposed bees [36]. Hence, we ask if 670nm exposure can correct this using metrics established for respiration. Initially haemocyte numbers were counted after 5 days Imidacloprid exposure.

Imidacloprid exposure significantly reduced haemocyte numbers by 61% which was corrected by 670nm exposure (Fig 2A). Again, we ask how much light is needed to correct this deficit using the same timetable as for respiration. 1 min light exposure was sufficient to correct haemocyte number following pesticide exposure (Fig 2B), and this was sufficient to significantly elevate cell numbers for 48h (Fig 2C).

**Fig 3:**
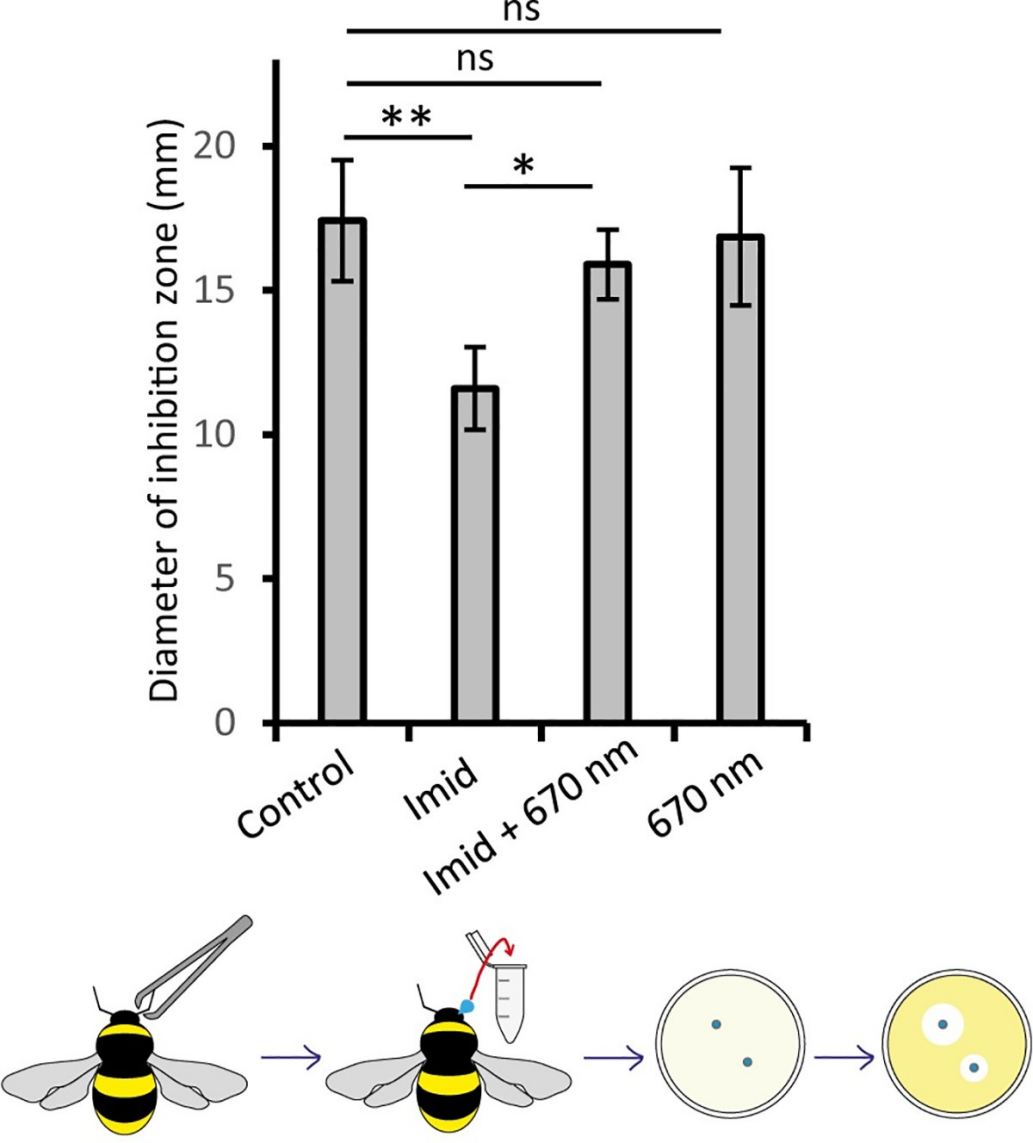
To confirm preservation of immunocompetency by 670nm, bumblebees were subjected to 4 day exposure of Imidacloprid and/or twice daily 670 nm prior to immune challenge with *E*.*coli*. Haemolymph was collected 24h later from antenna base and used in inhibition zone assays as in the schematic. Antimicrobial impact of haemolymph was used as a metric. After *E*.*coli* challenge, the haemolymph inhibited bacterial growth. The immunocompetence of haemolymph is significantly reduced after Imidacloprid exposure and partially restored with 670nm exposure after exposure. Abbreviations: *; p < 0.05, **; p < 0.01, ns; no significance, Imid; Imidacloprid. Bumblebees, ≥ 17 bees per group.

To confirm that Imidacloprid damage to immunocompetence could be rescued by 670nm exposure, the anti-microbial activity (lytic activity) of haemolymph was determined *ex-vivo* 24h after bees were injected with dead bacteria. Injecting dead bacteria exposes potential vulnerability in immunocompetence and is used here as a platform for the impact of Imidacloprid. The metric used is the size of the inhibitory zone around the haemolymph on a bacterial dish measured similar to Brandt et al. [36]. Haemolymph anti-microbial activity was significantly reduced in Imidacloprid exposed bumblebees following insecticide exposure (Fig 3). The size of the inhibition zone decreased by 33%. However, this was corrected in bumblebees exposed to 670nm light. Hence, both in terms of haemocyte number and their immunocompetence, Imidacloprid exposure had a significant negative impact, but was corrected by 670nm exposure (Fig 2). Additionally, challenging the immune system of the bee leads to attrition within the 24 h immune response period [55]. In the control group, 35% of the original cohort died, 50% in the Imidacloprid group died, whilst there was no deaths post immune challenge within 24h, in the two groups exposed to 670nm light.

## Discussion

We show that Imidacloprid undermines bumblebee respiration and immunocompetence and that this can be rescued by 670nm exposures of 1min. This positive impact lasts for 4–6 days in both cases and implies that respiration and immunocompetence have common underlying mechanisms that likely resides with mitochondrial integrity. These metrics and their response to 670nm light have not been examined previously and are very different from those examined in our initial study [33]. While there are an increasing number of pesticides available, it is likely that any targeting mitochondria will respond positively to 670nm light.

We have shown previously that 670nm light exposure in Imidacloprid exposed bumblebees restores damaged mitochondrial function improving mobility and lifespan [33]. But the issue of respiration and immunity have not been examined. As there is a complex relationship between mitochondria and immunity [56–58], 670nm could act on each independently or via their relationship. The nicotinic acetylcholine receptors, those with affinity to Imidacloprid, are present on honey bee haemocytes [59]. Haemocytes are likely to be responding to Imidacloprid directly, independently from the neural effects seen in our previous publication [33], although a neural pathway cannot be ruled out. The decrease in haemocyte numbers observed could be the result of apoptosis. In molluscs, blockage of muscarinic acetyl choline receptors promotes expression of tumor necrosis factor and subsequent apoptosis of haemocytes [60, 61]. As first proposed by Goulson [59], if a similar mechanism involving nicotinic acetylcholine receptors exists in bees, this could explain the decrease in haemocyte counts after Imidacloprid exposure, and this idea would be supported by restoration by 670 nm light. 670 nm light is known the prevent apoptosis [62], 670nm could therefore have a restorative effect on immunocompetency by reducing pesticide induced haemocyte apoptosis. There are two populations of haemocytes found in insects, circulating haemocytes (those measured in our study), and sessile haemocytes, those attached to tissues [63]. It is also possible that increased circulating cell numbers could be due to displacement of sessile haemocytes due to 670nm light, which then increase circulating numbers explaining the result seen. The preservation of haemocytes from undergoing apoptosis, or increased number due to detachment of sessile haemocytes, either instance would also explain the increased lytic activity of the haemolymph. Preserved haemocyte numbers account for the larger inhibition zones, as more anti-microbial peptides could be secreted into the haemolymph in response to immune challenge [64]. The immune challenge model employed triggers an immune response from both exposure to bacterial antigens, and wounding resulting from the injection [55]. In this instance we do not know which is predominating, however we have shown that Imidacloprid decreases the antimicrobial activity of the haemolymph, and that 670 nm light can restore immunocompetency. We see no reason as to why it could not be effective in either instance separately, however, we do show that with both triggers combined, the treatment is effective.

Gaps in understanding remain. We counted total haemocytes, but these are a heterogeneous population, and it is unclear which responds to the light [65]. Likewise, the restoration of cell number does not indicate restoration in immune system profile. Others have examined the effect of Imidacloprid on bumblebee immunity [34, 66] and changes in specific components of the immune response have been reported, including decreased phenoloxidase activity leading to melanisation [66] and encapsulation [36]. Pesticide exposure also results in inhibition of NF-κB signaling which plays a role in immunity in honey bees [37]. The expression of immune system related genes also changes with neonicotinoid exposure and the relationship between changes in gene expression and immunity remains to be largely explored [67].

Respiratory changes to 670nm light are similar to those in aged *Dropsophila*, where single exposures remained effective for 100 h [50]. Our data also show that exposures increased respiration in treated and untreated bumblebees above controls. It may be argued that this is problematic, but these exposures were associated with deficit correction following Imidacloprid exposure and increased lifespan [33]. Consequently, it is unlikely that they are directly detrimental overall. However, there may be other changes as a result of this elevated respiration that we have not explored and that may be detrimental. It is likely to be associated with a significant increase in metabolism that carries a cost. But as we do not know which tissues are most effected, it is difficult to evaluate the cost benefit ratio. It has been argued that deep red light is most effective on tissues with a high metabolic demand and greater mitochondrial density [68]. This would implicate the nervous system and the musculature, but less so other tissues where mitochondrial density is relatively low. However, because mitochondrial density is low, it does not mean that such tissues would not absorb long wavelengths via different mechanisms that would not impact on mitochondrial performance. In experiments not reported here, individual bumblebees have the potential to block >90% of the transmission of the 670nm LEDs we have used in this study when placed immediately adjacent to them, although in this situation there is a likely heat effect. Critically, we do not know which mitochondria are absorbing red light, in which tissues and how this is shifting their function. Further, we do not know the stream of events that result in improved longevity and how this might impact on a bee colony if its age structure is shifted towards an older group. These remain matters for subsequent exploration.

Weinrich et al. [45] revealed metabolic changes with 670nm in aged *Drosophila*, showing generally improvements that associates with robustness when challenged by chill, which is a stress factor for bees due to climate change. This study also showed improved memory, mobility, and retinal function that are all critical to stress challenges that bees face.

The debilitating impact of Imidacloprid and its correction by 670nm finds symmetry in many mammalian studies where 670nm has been employed to reduce induced CNS pathology, particularly that associated with mitochondrial insult [68, 69]. However, particularly pertinent to 670nm benefits in bees is the finding that old flies lose stereotypic navigation patters in open environments, but this improved after single 670nm exposures [50]. Hence, this light may help bees maintain their navigational abilities when foraging. As 670nm light also improves memory and retinal function [31, 33], its application to beehives/colonies may offer significant advantages.

A key feature of our data is the absence of any conventional dose dependent effects. Relatively long exposure to 670nm was not better than 1 min. This appears to be a feature of long wavelength light in mammalian models of induced pathology where variable light exposures between studies have similar impact [68]. Likewise, the effect of the light did not decline gradually but appeared to terminate over a short time. These data indicate the mechanism may be rather switch like. Further, the brief nature of effective exposures is potentially important because it may facilitate exposure to honey bees when passing through hive entrances. An additional advantage of 670nm light is that it is beyond the bee’s visual range and consequently they are not disturbed by it [70].

Longer wavelengths are present in daylight, and a natural question arises as to whether these may influence bee mitochondria. However, there is a key reason why this may not occur to any significant extent. The spectrum of daylight includes short as well as long wavelengths and shorter wavelengths are known to undermine mitochondrial function [71]. Hence, there is likely to be a complex interplay between these two wavelength ranges that may overall being their relative influence into approximate balance. While spectral components of daylight shift with time of day, changes in weather, environmental pollution, and a multitude of other factors, we do not know how their influence over mitochondria is integrated over time. If integration is over a longer period, it is possible that such shifts in environmental light could be largely ironed out. But there are too many variables in this complex interactive problem to cast light on our data. An additional complexity is that long wavelength exposure appears to be only effective earlier in the day, while we know of no similar evidence for short wavelengths [72]. How this may or may not relate to environmental light is unclear.

A further consideration in the context of long and short wavelengths is whether they directly stimulate cell function via the retina and if this drives changes in mitochondria. This can be excluded for 670nm light because the visual range of the bee does not extend beyond around 640nm and hence they are unable to see 670nm light [70, 73].

We have used bumblebees because colonies are commercially available year-round unlike honey bees. Further, bumblebee colonies are more suitable to the lab environment. However, we have used honey bees in limited respiration studies and their data are very similar to that presented here. Consequently, the effects we report and that are similar to the fly are likely to be similar in honey bees. Critically our experiments are lab based. However, Beefutures in France have adopted our technology in real field experiments and hence will provide the translation needed. Here, to date, the story is consistent with that in the lab, reinforcing the idea that long wavelength light is likely to be of value in sustaining this key pollinator.
